# Nuclear Cross-Section of Proton-Induced Reactions on Enriched ^48^Ti Targets for the Production of Theranostic ^47^Sc Radionuclide, ^46c^Sc, ^44m^Sc, ^44g^Sc, ^43^Sc, and ^48^V

**DOI:** 10.3390/ph17010026

**Published:** 2023-12-23

**Authors:** Liliana Mou, Lucia De Dominicis, Sara Cisternino, Hanna Skliarova, Matteo Campostrini, Valentino Rigato, Laura De Nardo, Laura Meléndez-Alafort, Juan Esposito, Férid Haddad, Gaia Pupillo

**Affiliations:** 1 Laboratori Nazionali di Legnaro (INFN-LNL), Istituto Nazionale di Fisica Nucleare, Viale dell’Università 2, Legnaro, 35020 Padova, Italy; liliana.mou@lnl.infn.it (L.M.); sara.cisternino@lnl.infn.it (S.C.); matteo.campostrini@lnl.infn.it (M.C.); valentino.rigato@lnl.infn.it (V.R.); juan.esposito@lnl.infn.it (J.E.); 2 Dipartimento di Fisica e Astronomia Galileo Galilei, Università degli Studi di Padova, Via F. Marzolo 8, 35131 Padova, Italy; laura.denardo@unipd.it; 3 INFN, Sezione di Padova (INFN-PD), Via F. Marzolo 8, 35131 Padova, Italy; hanna.skliarova@pd.infn.it; 4 Istituto Oncologico Veneto IOV-IRCCS, Via Gattamelata 64, 35138 Padova, Italy; laura.melendezalafort@iov.veneto.it; 5 GIP ARRONAX, 1 Rue Aronnax, 44817 Saint Herblain, France; ferid.haddad@subatech.in2p3.fr; 6 Laboratoire Subatech, IN2P3-CNRS, IMT Atlantique, Nantes Université, 4 Rue Alfred Kastler, 44307 Nantes, France

**Keywords:** ^47^Sc, ^48^Ti targets, cross-section measurements, proton cyclotron, nuclear reactions

## Abstract

The cross-sections of the ^48^Ti(p,x)^47^Sc, ^46c^Sc, ^44m^Sc, ^44g^Sc, ^43^Sc, and ^48^V nuclear reactions were measured from 18 to 70 MeV, with particular attention to ^47^Sc production. Enriched ^48^Ti powder was deposited on an aluminum backing and the obtained targets were characterized via elastic backscattering spectroscopy at the INFN-LNL. Targets were exposed to low-intensity proton irradiation using the stacked-foils technique at the ARRONAX facility. Activated samples were measured using γ-spectrometry; the results were compared with the data int he literature and the theoretical TALYS-based values. A regular trend in the new values obtained from the different irradiation runs was noted, as well as a good agreement with the literature data, for all the radionuclides of interest: ^47^Sc, ^46c^Sc, ^44m^Sc, ^44g^Sc, ^43^Sc, and ^48^V. ^47^Sc production was also discussed, considering yield and radionuclidic purity, for different ^47^Sc production scenarios.

## 1. Introduction

This work was carried out in the framework of the Production with Accelerator of Sc-47 for Theranostic Applications (PASTA) [1] and Research on Emerging Medical Radionuclides from the X-sections (REMIX) [2] projects, funded by INFN in 2017/2018 and 2021/2023, respectively. With the 70 MeV proton cyclotron installed at the INFN-LNL, the research activities carried out within Laboratory of Radionuclides for Medicine (LARAMED) are focused on the production of emerging radionuclides with proton beams [2]. Among the radionuclides of major interest for our team is ^47^Sc, a theranostic radionuclide that presents suitable decay characteristics for SPECT imaging and β^−^ therapy (Table 1), which can be also paired with the β^+^ emitter counterparts ^43^Sc and ^44^Sc for PET applications [3,4,5,6,7,8,9,10,11,12,13,14,15,16,17,18,19,20]. The lack of ^47^Sc production is limiting its use in preclinical and clinical trials; for this reason, the ^47^Sc proton-based production routes have been investigated within the PASTA and REMIX projects [1,2]. First, ^nat^V targets have been considered [21,22], then the nuclear reactions induced on isotopically enriched ^48^Ti, ^49^Ti, and ^50^Ti targets (natural abundances of 73.72%, 5.41%, and 5.18%, respectively [23]) were studied. This paper presents the new data obtained for the ^48^Ti(p,x)^47^Sc, ^46c^Sc, ^44m^Sc, ^44g^Sc, ^43^Sc, and ^48^V cross-sections, which we compared with the literature data available on the EXFOR database [24,25,26] and the TALYS estimations [27,28,29]. The results include the ^46c^Sc cumulative cross-section, due to the production of ^46g^Sc with a half-life of 83.79 d, and ^46m^Sc, which has a half-life of 18.75 s and decays to ^46g^Sc. Table 1 reports the main decay characteristics of the radionuclides studied in this work, as extracted from the NuDat 3.0 database [23].

The literature on proton-induced reactions with Ti-enriched targets is scarce; Gadioli et al. [30] and Levkovski [31] published data, respectively, in 1981 and 1991, using enriched ^48^TiO_2_ samples. Mausner et al. (1998) measured the relative cross-sections of ^46c^Sc, ^44m^Sc, ^48^Sc normalized to ^47^Sc, in the energy range 48–150 MeV using enriched ^48^TiO_2_ targets (99.81%); however, it is not possible to extract these absolute cross-section values [32]. Some experimental data are also available for the ^48^Ti(p,n)^48^V cross-section by rescaling the low energy (p,n) values obtained with ^nat^Ti targets for the case of fully enriched material [24].

Enriched metallic ^48^Ti powder was used in this work and deposited with the high energy vibrational powder plating (HIVIPP) technique [33,34], developed within the E_PLATE project (INFN in 2018/2019), on a substrate [35,36]. A complete characterization of the ^48^Ti-enriched targets was performed with the elastic backscattering (EBS) method using the proton beam available at the AN2000 accelerator at INFN-LNL. The EBS technique allowed the measurement of the amount of ^48^Ti deposited (µg/cm^2^) and its homogeneity. The nuclear cross-section measurements were performed at the ARRONAX facility [37], exploiting the available proton beam with tunable energy ranging from 35 to 70 MeV.

## 2. Discussion and Results

The nondestructive EBS technique was used to quantify the composition, the Ti deposited amount in µg/cm^2^, and the lateral homogeneity of the manufactured ^48^Ti-enriched targets. The spectra acquired on the same target at three different points can overlap; the corresponding values of the Ti amount are thus similar, and the uniformity of the depositions along the diameter is therefore confirmed by the EBS analysis. The final value of the thickness in µg/cm^2^ used for the nuclear cross-section calculations is the mean of the values measured at the three points. The mean value and the standard deviation for each sample are reported in Table 2. The targets prepared using the HIVIPP technique presented no modification after irradiation; the ^48^Ti deposit remained adherent to the Al substrate.

**Figure 1:**
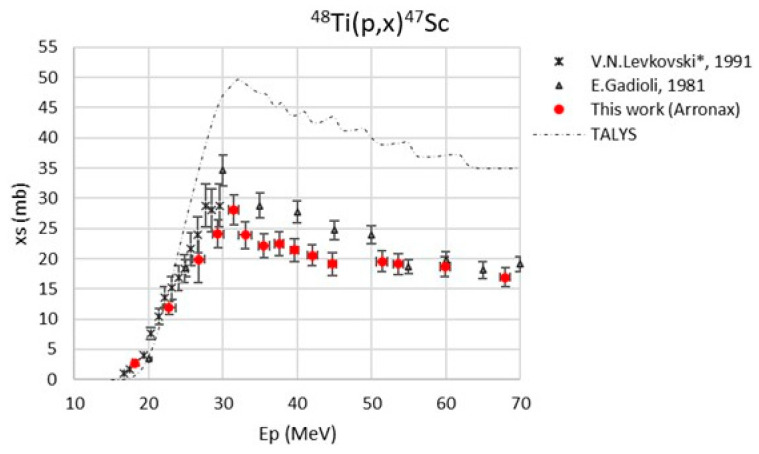
Cross-section of the ^48^Ti(p,2p)^47^Sc nuclear reaction [30,31].

**Figure 2:**
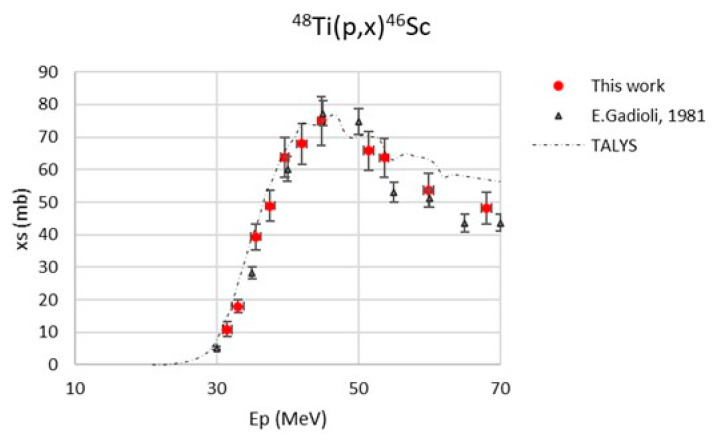
Cross-section of the ^48^Ti(p,x)^46c^Sc nuclear reaction [30].

**Figure 3:**
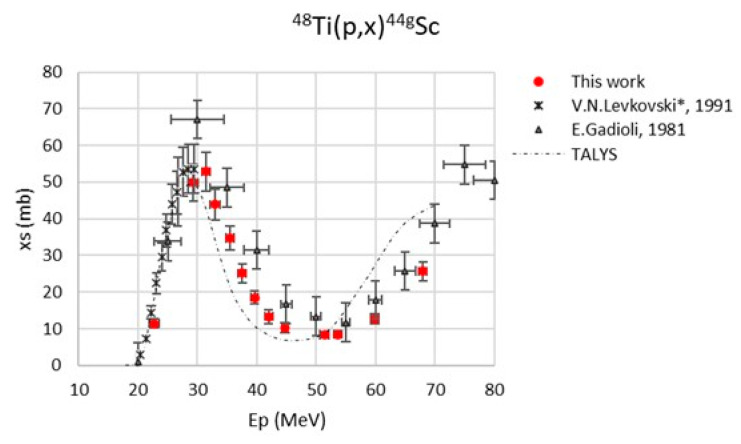
Cross-section of the ^48^Ti(p,x)^44g^Sc nuclear reaction [30,31].

**Figure 4:**
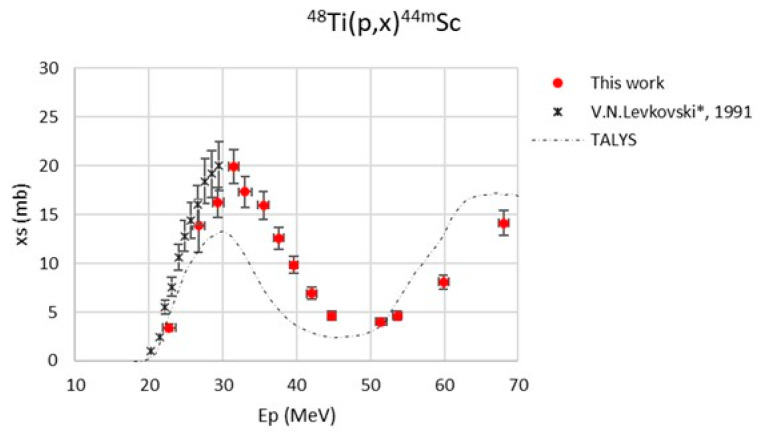
Cross-section of the ^48^Ti(p,x)^44m^Sc nuclear reaction [31].

**Figure 5:**
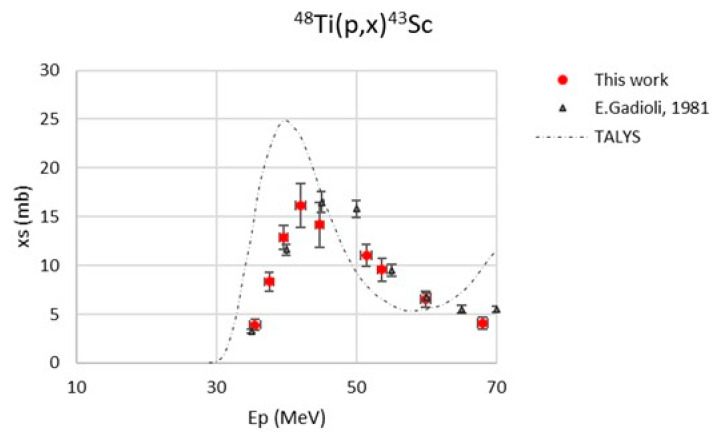
Cross-section of the ^48^Ti(p,x)^43^Sc nuclear reaction [30].

The maximum value of the beam energy uncertainty, calculated with SRIM2003 code [38], was 875 keV. The major contribution to the cross-section uncertainty was always the monitor cross-section (max. 5%) [39,40]. The monitor reaction values used in the data analysis are reported in Table 3. The new experimental cross-section values, referring to a 100% enriched ^48^Ti target, are reported in Table 4 and are plotted in Figure 1, Figure 2, Figure 3, Figure 4 and Figure 5. A comparison with the literature data and the TALYS results (represented with a dashed line) is also given [29]. TALYS simulations were performed using default parameters; additional information on the reaction, level density, and optical models used by the TALYS code can be found in [27,28,41]. The results show a regular trend for all six radionuclides, ^47^Sc, ^46^Sc, ^44g^Sc, ^44m^Sc, ^43^Sc, and ^48^V.

**Figure 6:**
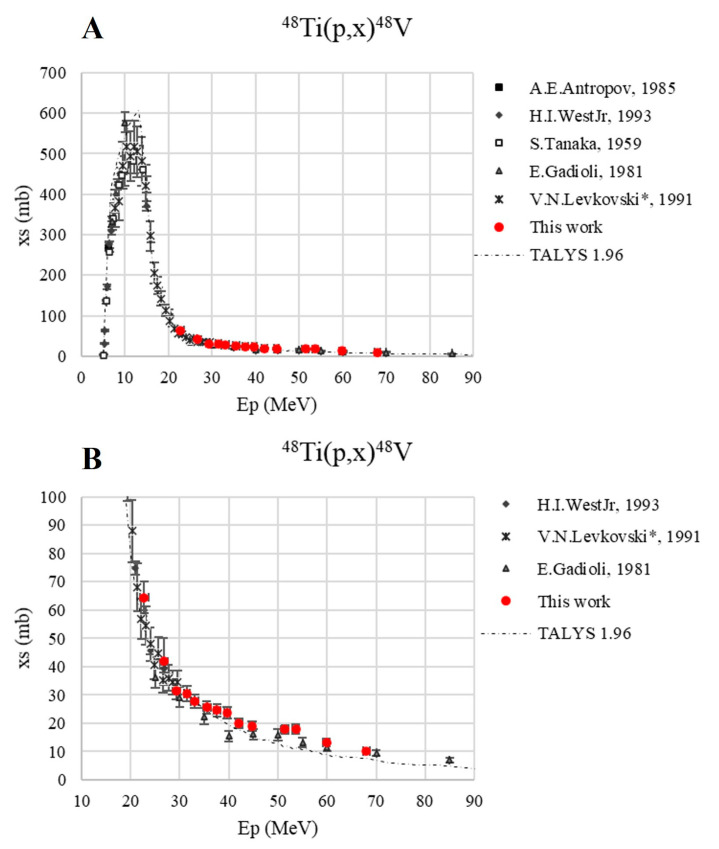
Cross-section of the ^48^Ti(p,x)^48^V nuclear reaction [24,25,30,31] up to 90 MeV (**A**) and in the energy range of interest, 20–70 MeV (**B**).

The data by Levkovski were corrected by a factor of 0.8 due to the monitor values used in 1991 [42]; for this reason, the data presented in the plots (Figure 1, Figure 3, Figure 4, and Figure 6) have a star in the legend to indicate the applied rescaling factor. There is a general good agreement of our new results with the literature data, even though our experimental values are about 20% lower than the data measured by Gadioli et al. (1981) in the energy range between 32 and 50 MeV [30].

A possible explanation could be the targets (e.g., enrichment level, composition, and target manufacturing) or on the monitor reactions and decay data used. The enrichment level of the target material was 99.1% for Gadioli et al., and that for Levkovski ranged from 95% to 98%. Gadioli et al. mixed ^48^TiO_2_ powder with ^nat^CuO (in a 2:1 ratio) to monitor the beam intensity with ^63^Cu(p,n)^63^Zn and ^65^Cu(p,x)^64^Cu reactions, respectively, up to 20 MeV and for the 25–85 MeV energy range. Nowadays, the IAEA recommends monitoring the ^nat^Cu(p,x)^63^Zn reaction up to 100 MeV [39,40]; thus, it is possible to rescale the IAEA data for the low-energy region, up to the ^63^Cu(p,n)^63^Zn channel (E_P_ < 22 MeV), and to compare the actual monitor values with the ones reported by Gadioli et al. 40 years ago. Up to 20 MeV, there is a very good agreement on the monitor reaction values, since the discrepancy is lower than 5%. However, the IAEA does not recommend the ^65^Cu(p,x)^64^Cu or the ^nat^Cu(p,x)^64^Cu reactions. It is worth noting that the literature data were obtained considering old values for the decay characteristics for the radionuclides of interest (discrepancies of up to 5%); however, it is not possible to correct these cross-section data considering the present values reported in Table 1 or to predict if the eventual correction would lead to an increase or decrease of the published values in 1981 and 1991.

Figure 2 presents the ^48^Ti(p,x)^46c^Sc cross-section measured up to 70 MeV, which is compared with the data of Gadioli et al. [30] and TALYS results. There is a general very good agreement on the description of the ^48^Ti(p,x)^46c^Sc nuclear reaction in the entire energy range investigated.

Figure 3 reports the ^48^Ti(p,x)^44g^Sc cross-section: there is a perfect agreement with the results obtained by Levkovski up to 30 MeV [31]; at higher energies, the new data are about 30% lower than the values measured by Gadioli et al. [30]. In general, our new values seem to be in agreement with the trend described by the TALYS estimations, which however present a slight energy shift toward lower energies, especially for E_P_ > 50 MeV.

The ^48^Ti(p,x)^44m^Sc cross-section is plotted in Figure 4: there is a general agreement with the results obtained by Levkovski [31], while the TALYS results seem to underestimate the peak at around 30 MeV by a factor of two, but the general trend in this nuclear reaction is properly described.

Figure 5 reports the ^48^Ti(p,x)^43^Sc excitation function: a good agreement with the results obtained by Gadioli et al. [30] can be noted. In this case, the TALYS results present a visible energy shift and a general overestimation of the cross-section.

Figure 6 reports the ^48^Ti(p,x)^48^V cross-section up to 90 MeV (A) and in the energy range investigated in this work, i.e., 20–70 MeV (B). There are several experimental data available on the EXFOR database [24,25], and our new values are in very good agreement both with the estimations in the literature and those of TALYS, which properly describe the reaction even if an overestimation of the peak value at ca. 13 MeV can be noted.

## 3. Discussion on ^47^Sc Production

**Figure 7:**
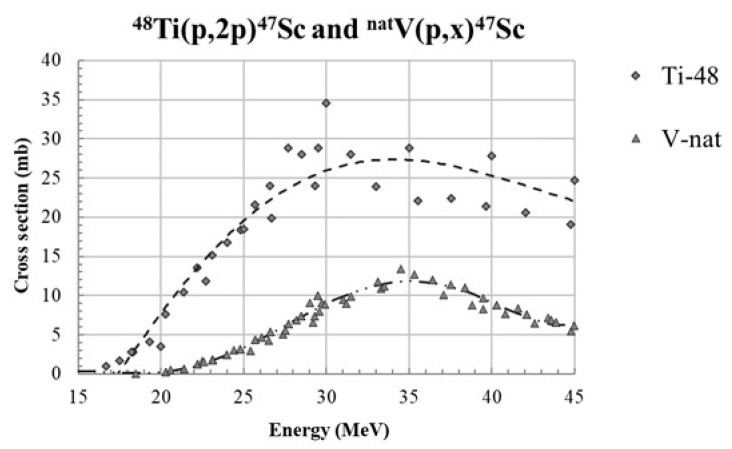
Comparison of the p-induced cross-sections for ^47^Sc production on ^48^Ti and ^nat^V targets. The dashed lines represent the fit of all the experimental data for both production routes.

The new cross-section data provided in this paper can be compared with the proton-based production route investigated with ^nat^V targets [1,21,22], especially in the energy region below 45 MeV, where the ^47^Sc cross-sections present a peak value, as shown in Figure 7. It can be noted that the ^47^Sc excitation function for ^48^Ti targets is larger than the one induced with ^nat^V targets. As previously reported, in the energy range up to 30 MeV, the ^47^Sc yield with ^nat^V targets is calculated as 41.5 MBq/μA and 111 MBq/μA for 24 h and 80 h irradiation runs, respectively [1]; on the other hand, considering enriched ^48^Ti targets, the ^47^Sc yield for E_P_ < 30 MeV is ca. 200 MBq/μA and 530 MBq/μA for 24 h and 80 h, respectively. From these calculations, performed using the ISOTOPIA tool [43] made available by the IAEA, it can be inferred that ^47^Sc production is about five times larger when using ^48^Ti targets instead of ^nat^V.

Particular attention has to be paid to the coproduction of Sc contaminants, i.e., ^46^Sc, ^44m^Sc, ^44g^Sc, and ^43^Sc, whose contribution in the final ^47^Sc-labeled radiopharmaceutical has to be carefully assessed for each energy range considered. Table 5 reports the Sc radionuclide activities produced in different scenarios considering proton beams and enriched ^48^Ti targets; in the calculation, all the experimental data available from the EXFOR database and the new ones measured in this work were considered to fit the nuclear cross-sections. Radionuclidic impurities can have undesirable effects on the patient’s overall radiation dose, as well as on the image quality, so the European Pharmacopoeia established limits of radionuclides impurities for each radiopharmaceutical in the individual monographs to guarantee safe clinical application [44]. In general, this limit is set to lower than 1%, but if very-high-purity products are technically achievable, it can be drastically reduced, up to 0.1% [45].

**Figure 8:**
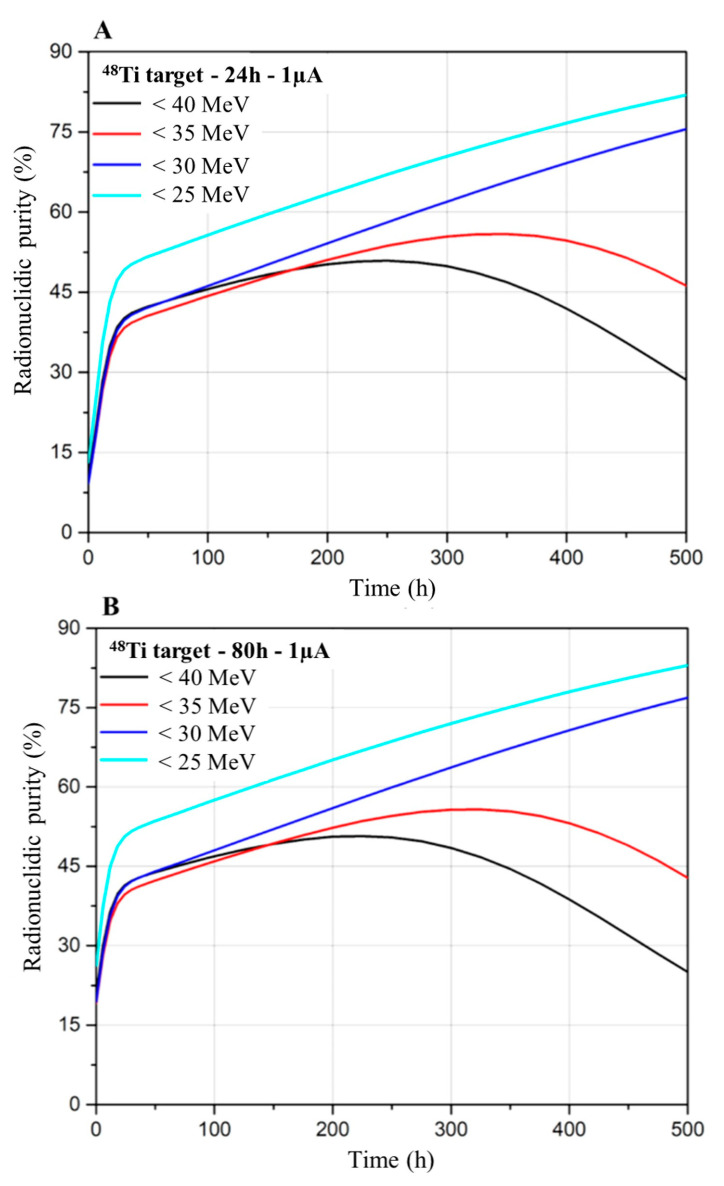
Comparison of the ^47^Sc RNP for 24 h (**A**) and 80 h (**B**) irradiation, using proton beams and ^48^Ti targets.

Figure 8 shows the radionuclidic purity (RNP) of ^47^Sc considering ^48^Ti targets and different scenarios for 24 h (A) and 80 h (B) irradiation, respectively. For all the cases, the RNP initially rapidly increases due to the decay of the short-half-time ^43^Sc and ^44g^Sc impurities. After about 30 h, the rise in the RNP occurs more slowly due to the decay of ^44m^Sc.

For irradiations performed at energies equal or larger than 35 MeV, the RNP reaches a maximum (of the order of 50 and 55%, for E < 40 MeV and E < 35 MeV, respectively) and then it decreases due to the contribution of the long-half-life ^46^Sc impurity. For lower-beam-energy irradiations (i.e., E_P_ < 30 MeV), the RNP instead continuously increases since, in these scenarios, ^46^Sc is not produced. The limit of ^47^Sc RNP = 99% can be reached about 1500 h after the EOB, corresponding to almost 20 times the half-time of ^47^Sc. It can be concluded that the use of ^48^Ti targets provides, at the EOB, a larger ^47^Sc yield than ^nat^V targets but with a much lower RNP [22].

## 4. Materials and Methods

### 4.1. Enriched ^48^Ti Targets

**Figure 9:**
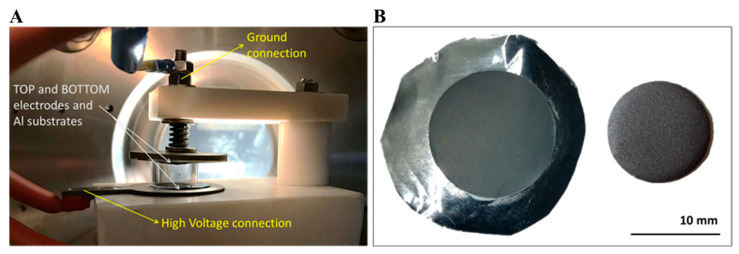
Photograph of the HIVIPP set up inside the vacuum chamber (**A**). Picture of the ^48^Ti-7 sample, with a typical ^48^Ti deposition onto Al, as realized and after punching (**B**).

Thin deposits of enriched ^48^Ti metallic powder (99.32%, purchased from Trace Sciences International Inc., Wilmington, DE, USA) onto a natural high-purity Al foil (99%, 25 µm thick, Goodfellow Cambridge Ltd., Huntingdon, UK) were obtained using the HIVIPP technique [35,36]. Briefly, the deposition process was based on the application of an electrostatic field of 15 kV/cm between two Al substrates, used as electrodes, to start the superficial charging and the motion of the powder closed inside a quartz cylinder. The process took place in a vacuum of about 1∙10^−7^ mbar and lasted about 30 h. Figure 9A shows the experimental set up of HIVIPP deposition used in this study. The ^48^Ti deposit had a diameter of 14 mm, which was cut then with punches (diameter of 12 mm) to fit the target holder used for the irradiation runs. A typical target foil is shown in Figure 9B. The peculiarities of this technique are the possibility of (i) realizing two substrates simultaneously, for which the target areal thickness of 0.2–2 mg/cm^2^ was achieved, and (ii) recovering undeposited enriched ^48^Ti powder, limiting the losses of this expensive material. More details about the technique and HIVIPP deposit characteristics are described in Refs. [35,36]. The EBS analysis on ^48^Ti targets was performed at the AN2000 Van the Graaff accelerator using a collimated 1800 keV proton beam with an approximate size of 1 mm^2^. The backscattering angle (θ^out^) and the incidence angle with respect to sample normal (θ^o^) were θ^out^ = 160° and θ^o^ = 0°. The measurements were made using a standard charged particle spectroscopy system consisting of a Si detector and NIM electronics.

**Figure 10:**
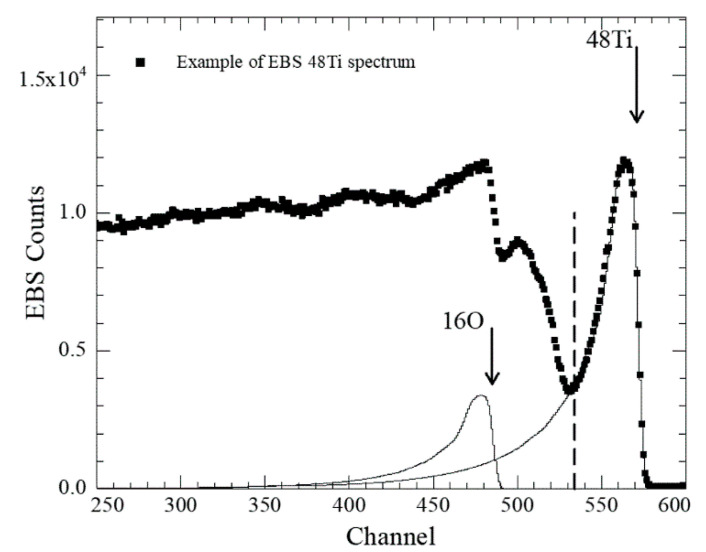
Experimental spectrum of ^48^Ti coating deposited onto Al substrate. Arrows indicate the ^16^O and ^48^Ti contributions.

The total energy resolution of the spectrometer was 13 keV. To ascertain coating uniformity, EBS measurements were performed for at least three positions on each sample along the sample’s diameter. The aluminum backings were also characterized using proton induced X-ray emission (PIXE) analysis to determine the presence of impurities. It turned out that 0.4 at% (±0.1) Fe was present in the Al substrates. The EBS experimental spectra were simulated using SimNRA 7.03 software [46]. The individual elements’ stopping powers were deduced from SRIM2003 code [38], and Bragg’s rule was used for the compounds. The non-Rutherford oxygen and aluminum backscattering cross-sections were deduced from the IAEA Ion Beam Analysis Nuclear Data Library database [47]. All other heavier relevant elements were assumed to have Rutherford cross-sections. The simulations took into account the significant coating roughness, which determined a long tail of the ^48^Ti signal toward the low-energy region of the spectra [48], as shown in Figure 10.

Simulation parameters were chosen to allow spectral fitting of the elements characterized by elastic scattering cross-section with a fine structure [49]. In all the analyzed samples titanium resulted oxidized. The determination of the ^48^Ti content was estimated by considering the Ti EBS simulated spectrum made of two contributions: the high energy part (characterized by low measurement error) and the Ti spectrum region tailing into the lighter elements, to which a higher uncertainty must be attributed due to the errors of the stopping powers and of the non-Rutherford cross sections. The results of the simulations are reported in Table 2.

### 4.2. Irradiation Runs, γ-Spectrometry, and Data Analysis

Fifteen ^48^Ti targets, assembled with the well-known stacked-foils technique, were irradiated in eight irradiation runs at the ARRONAX facility [37] to cover the energy range of 18–70 MeV. The irradiation runs had a duration of 50–90 min, with a current of about 100–130 nA, monitored during the bombardment using an instrumented beam dump. The beam line was under vacuum, closed with a 75 µm thick Kapton foil; the stacks were located about 10–15 cm downstream in air; this distance was precisely measured for each irradiation run.

**Figure 11:**
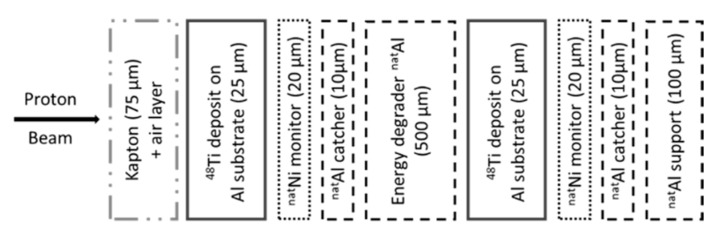
Scheme of a typical SRIM calculation for the stacked-foil target.

Close to each ^48^Ti target, a ^nat^Ni monitor foil was inserted (10 or 25 μm thick) in order to measure the effective beam flux by considering the reference reaction recommended by the International Atomic Energy Agency (IAEA) [39,40] for ^57^Ni production (half-life 35.60 h, E_γ_ = 1377.63 keV, I_γ_ = 81.7%). To catch the eventual ^57^Ni recoil atoms, a thin aluminum foil (10 μm thick) was inserted after the ^nat^Ni monitor foil. To decrease the beam energy, some thicker Al foils (500 μm thick) were used in the stacked structure, as shown in Figure 11. All the materials used in the stacks were high-purity foils (≥99%, Goodfellow Cambridge Ltd., Huntingdon, UK).

The proton beam energy in each layer of the stacked target was calculated using SRIM2013 code [50], considering the extracted proton beam energy from the cyclotron, and the energy losses in the Kapton foil, in the air and in each foil of the stacked targets. The uncertainty on the proton beam energy was obtained by considering the uncertainty of the energy extracted from the cyclotron (±500 keV) and calculating the energy straggling through each layer of the stacked target using SRIM.

Given that the ^48^Ti powder was deposited on an Al substrate, the stacked-foil structure was always assembled in order to have the proton beam impinging on the ^48^Ti powder first; in this way, recoil atoms were trapped by the Al support.

**Figure 12:**
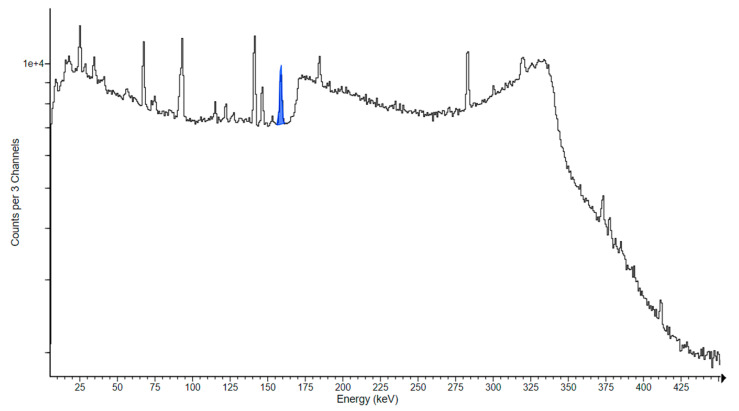
Typical γ spectrum obtained with an HPGe detector for an irradiated ^48^Ti target. In blue, the ^47^Sc γ-peak is highlighted.

As soon as possible after the end of bombardment (EOB), a first γ-spectrometry measurement of the irradiated ^48^Ti sample was carried out to estimate the activity of short-living radionuclides: this acquisition was typically 15 min long, and it was performed about 2–4 h after the EOB. Each ^48^Ti sample was also measured overnight to check for lower-activity products with a longer acquisition time (about 8–14 h). To follow the decay of the radionuclides of interest and to check for eventual γ-interferences, the γ-spectrometry measurements were repeated for all ^48^Ti targets each day up to 5 days after the EOB (these acquisitions were typically 1.5–3 h long). All samples were measured with the same high-purity germanium (HPGe) detector (10% relative efficiency, FWHM 1.0 keV at 122 keV, Canberra GC1020), previously calibrated with a ^152^Eu and an ^241^Am point-like solid sources (purchased to Cerca-Lea, Tricastin, France). All ^48^Ti samples were measured with the ^48^Ti deposit in the direction of the HPGe detector in order to avoid the γ attenuation due to the Al support. The sample–detector distance was fixed at 19 cm to reduce the dead time during measurements, which was always kept below 10%. The γ spectra were analyzed using software jRadView, developed at the INFN-LNL for nuclear physics experiments. A typical γ spectrum obtained for a ^48^Ti target is shown in Figure 12.

The nuclear data extracted from the NuDat 3.0 database (Table 1) were used in the data analysis, which was carried out following the methods of Otuka et al. [51], which were also used for the uncertainty calculations. In the calculation of the ^46c^Sc cumulative cross-section, only the γ line at 889 keV was used, since the 1120 keV line had an interference with the background ^214^Bi emission from the natural ^238^U decay chain. The recoil effect for the monitor ^57^Ni activity was taken into account, and it was about 1%. The results of the ^48^Ti(p,x)^47^Sc, ^46c^Sc, ^44m^Sc, ^44g^Sc, ^43^Sc, and ^48^V cross-sections are given for a 100% enriched target. New data were compared with the few experimental values available and with the results obtained from the TALYS code run with the default parameters (version 1.96 released in December 2021) [29].

## 5. Conclusions

The new cross-section data provided in this paper for the ^48^Ti(p,x)^47^Sc, ^46c^Sc, ^44m^Sc, ^44g^Sc, ^43^Sc, and ^48^V reactions are generally in a good agreement with the literature data. The TALYS results give a satisfactory description of the trend in the nuclear reactions (e.g., ^46c^Sc and ^44g^Sc), even if there is a considerable energy shift and an overestimation of the experimental values in the case of ^43^Sc. Theoretical studies to find the best TALYS parameters to properly describe the nuclear reactions are ongoing in the framework of the REMIX collaboration [21,52].

The calculations showed that the ^48^Ti(p,x)^47^Sc route provides a larger ^47^Sc yield compared to the use of ^nat^V targets, but with an RNP not suitable for medical applications. To provide a comprehensive overview of the proton-induced routes for ^47^Sc production, nuclear cross-section measurements using enriched ^49^Ti and ^50^Ti targets are ongoing within the REMIX project. These new data will be compared with those based on the use of ^nat^V and ^48^Ti targets to select the most suitable irradiation parameters for a reliable ^47^Sc supply.

## Figures and Tables

**Table 1:** Nuclear data associated with the radionuclides studied in this work [23]; the uncertainty is reported in brackets.

	Half-Life	γ-Ray Energy (keV)	γ-Ray Intensity (%)	Mean β^−^ Energy (keV)	Total β^−^ Intensity (%)	Mean β^+^ Energy (keV)	Total β^+^ Intensity [%]
^47^Sc	3.3492 d (6)	159.381 (15)	68.3 (4)	162.0 (21)	100.0 (8)		
^46^Sc	83.79 d (4)	889.277 (3) 1120.545 (4)	99.9840 (10) 99.9870 (10)	111.8 (3)	100.0000 (10)		
^44m^Sc	58.61 h (10)	271.251 (10) 1157.002 (15)	86.72 1.23				
^44g^Sc	4.0420 h (425)	1157.022 (15)	99.8867 (30)			630.2 (8)	94.278 (11)
^43^Sc	3.891 h (12)	372.9 (3)	22.5			476 (6)	88.1 (8)
^48^V	15.974 d (3)	983.525 (4) 1312.105 (6)	99.98 (4) 98.2 (3)			291.4 (25)	50.4 (3)

**Table 2:** Results of the EBS analysis on the enriched ^48^Ti samples.

Target ID	^48^Ti Deposit (µg/cm^2^)	Target ID	^48^Ti Deposit (µg/cm^2^)
^48^Ti-01	190 ± 11	^48^Ti-09	524 ± 26
^48^Ti-02	292 ± 10	^48^Ti-10	183 ± 11
^48^Ti-03	501 ± 17	^48^Ti-11	259 ± 11
^48^Ti-04	190 ± 11	^48^Ti-12	590 ± 26
^48^Ti-05	292 ± 10	^48^Ti-13	524 ± 26
^48^Ti-06	674 ± 32	^48^Ti-14	674 ± 32
^48^Ti-07	590 ± 26	^48^Ti-15	520 ± 25
^48^Ti-08	520 ± 25		

**Table 3:** IAEA monitor cross-section values for the ^nat^Ni(p,x)^57^Ni reaction [39].

Energy (MeV)	^57^Ni (mb)	Energy (MeV)	^57^Ni (mb)
22.4	148.7 ± 6.3	42.2	80.4 ± 3.5
26.3	180.1 ± 7.5	44.6	77.8 ± 3.4
29.0	162.8 ± 6.8	51.3	73.4 ± 3.1
31.3	134.0 ± 5.6	53.5	72.3 ± 3.1
33.1	116.6 ± 4.9	59.7	69.5 ± 3.0
35.6	99.3 ± 4.2	67.9	66.2 ± 2.9
39.3	85.6 ± 3.6		

**Table 4:** Measured cross-sections for the ^48^Ti(p,x)^47^Sc, ^46c^Sc, ^44g^Sc, ^44m^Sc, ^43^Sc, ^48^V reactions in the 18–70 MeV energy range.

**Target ID**	**Energy (MeV)**	**^47^** **Sc** **(mb)**	**^46c^** **Sc** **(mb)**	**^44g^** **Sc** **(mb)**	**^44m^** **Sc** **(mb)**	**^43^** **Sc** **(mb)**	**^48^** **V** **(mb)**
^48^Ti-01	18.2 ± 0.4	4.0 ± 0.3	-	-	-	-	
^48^Ti-02	22.7 ± 0.9	11.9 ±1.1	-	11.5 ± 1.2	3.4 ± 0.3	-	64.5 ± 5.7
^48^Ti-03	26.7 ± 0.8	19.9 ± 3.9	-	47.4 ± 9.4	13.9 ± 2.7	-	41.9 ± 8.2
^48^Ti-04	29.3 ± 0.8	24.1 ± 2.3	-	49.9 ± 5.0	16.3 ± 1.6	-	31.4 ± 3.0
^48^Ti-05	31.5 ± 0.7	28.1 ± 2.5	10.9 ± 2.2	52.8 ± 5.2	19.9 ± 1.8	-	30.5 ± 2.7
^48^Ti-06	33.0 ± 0.8	23.9± 2.2	18.0 ± 2.0	43.9 ± 4.2	17.3 ± 1.6	-	27.8 ± 2.5
^48^Ti-07	35.5 ± 0.7	22.1 ± 2.0	39.3 ± 3.9	34.8 ± 3.3	16.0 ± 1.4	3.9 ± 0.6	25.6 ± 2.3
^48^Ti-08	37.6 ± 0.7	22.4 ± 2.0	48.9 ± 4.8	25.1 ± 2.5	12.6 ± 1.1	8.3 ± 0.9	24.6 ± 2.2
^48^Ti-09	39.6 ± 0.6	21.4 ± 1.9	63.7 ± 6.2	18.5 ± 1.8	9.8 ± 0.9	12.9 ± 1.2	23.7 ± 2.2
^48^Ti-10	42.0 ± 0.7	20.6 ± 1.8	67.9 ± 6.2	13.3 ± 1.9	6.9 ± 0.6	16.2 ± 2.2	19.9 ± 1.7
^48^Ti-11	44.8 ± 0.5	19.1 ± 1.8	75.0 ± 7.4	10.4 ± 1.4	4.6 ± 0.5	14.4 ±2.3	19.0 ± 1.8
^48^Ti-12	51.4 ± 0.8	19.5 ± 1.7	65.9 ± 6.0	8.4 ± 0.8	4.0 ± 0.4	11.0 ±1.1	17.8 ± 1.6
^48^Ti-13	53.6 ± 0.7	19.1 ± 1.7	63.7 ± 5.9	8.4 ± 0.9	4.6 ± 0.4	9.6 ± 1.2	17.9 ± 1.6
^48^Ti-14	59.9 ± 0.7	18.7 ± 1.7	53.7 ± 5.1	12.7 ± 1.3	8.1 ± 0.7	6.5 ± 0.8	13.2 ± 1.2
^48^Ti-15	68.0 ± 0.7	16.9 ± 1.5	48.2 ± 4.8	25.7 ± 2.6	14.1 ± 1.3	4.1 ± 0.6	10.1 ± 1.0

**Table 5:** Sc radionuclide yields calculated for several scenarios, considering 1 µA proton beam current and enriched ^48^Ti targets.

E_p_ on ^48^Ti Targets (MeV)	^47^Sc [MBq] (mCi)	^46c^Sc [MBq] (mCi)	^44g^Sc [MBq] (mCi))	^44m^Sc [MBq] (mCi)]	^43^Sc [MBq] (mCi)
T_irr_ = 24 h					
E_p_ < 25	73 (2)	-	447 (12)	39 (1)	-
E_p_ < 30	198 (5)	-	1792 (48)	154 (4)	-
E_p_ < 35	364 (10)	4.8 (0.1)	3270 (88)	297 (8)	19.9 (0.5)
E_p_ < 40	556 (15)	19.4 (0.5)	4232 (114)	412 (11)	289 (8)
T_irr_ = 80 h					
E_p_ < 25	196 (5)	-	454 (12)	96 (3)	-
E_p_ < 30	529 (14)	-	1820 (49)	381 (10)	-
E_p_ < 35	971 (26)	15.8 (0.4)	3320 (90)	736 (20)	20.2 (0.6)
E_p_ < 40	1481 (40)	64 (2)	4298 (116)	1019 (28)	293 (8)

## Data Availability

Data are contained within the article.
